# Supplementary material for: Counting rare Wolbachia endosymbionts using digital droplet PCR
Source: Microbiol Spectr. 2025 Apr 16;13(6):e03266-24. doi: 10.1128/spectrum.03266-24 (PMC12131755; doi:10.1128/spectrum.03266-24)
Supplement: Fig. S1 — HEX amplitude distribution in singleplex ftsZ-ddPCR assays. [file spectrum.03266-24-s0004.pdf]

Counting rare *Wolbachia* endosymbionts using digital droplet PCR

Alphaxand K. Njogu[1], Francesca Logozzo[1], William R. Conner[2], and J. Dylan Shropshire[1]

[1]Department of Biological Sciences, Lehigh University, Bethlehem, Pennsylvania, USA
[2]Division of Biological Sciences, University of Montana, Missoula, Montana, USA

Supplemental Figures

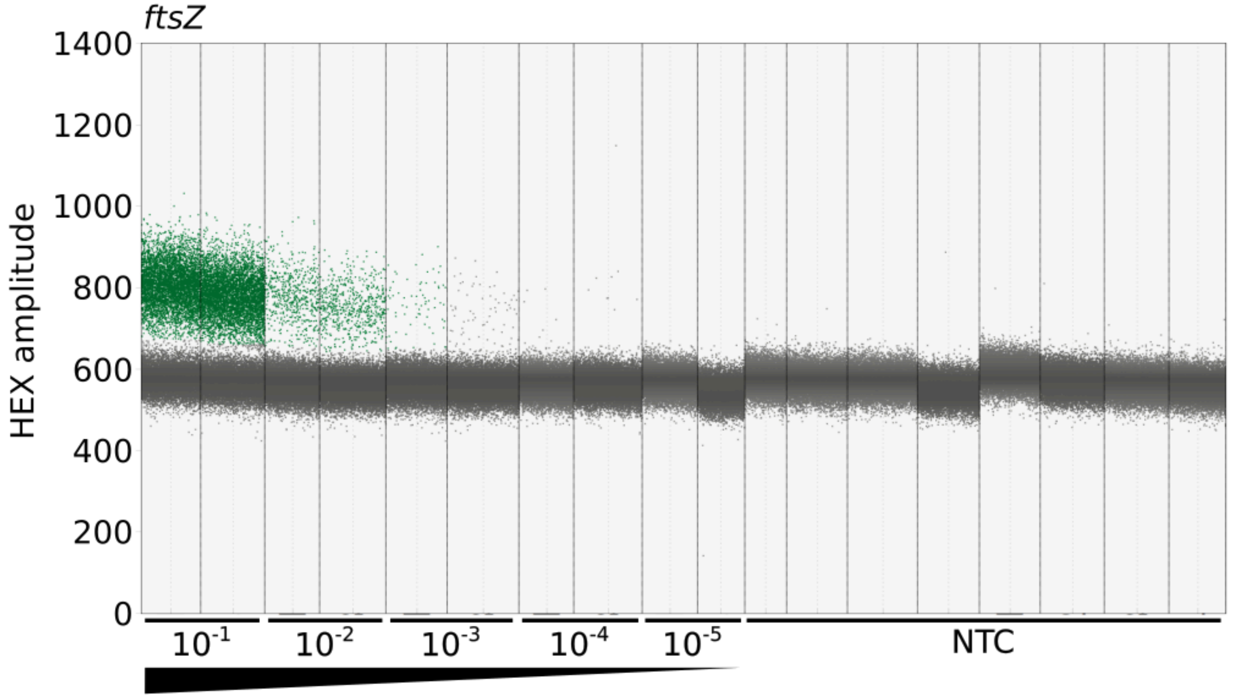

Figure S1. HEX amplitude distribution in singleplex *ftsZ*-ddPCR assays. Data is derived from the experiment presented in Figure 1D, specifically focusing on the HEX channel. Despite lacking HEX-labeled probes, HEX-amplitude varies between *ftsZ*-positive and *ftsZ*-negative droplets, indicating bleedthrough from the FAM to the HEX channel. Vertical dotted lines delineate the results from 20 μL ddPCR reactions, each containing 2 μL of DNA template. When multiple reactions were performed for a treatment condition, they were technical replicates derived from the same DNA extract. Data points on the plots represent individual droplets, with each reaction analyzed containing no fewer than 10,000 droplets. Droplets exhibiting high amplitude (green) indicate the presence of the target template, while low amplitude droplets (gray) represent the absence of the template.
