# Supplementary material for: Counting rare Wolbachia endosymbionts using digital droplet PCR
Source: Microbiol Spectr. 2025 Apr 16;13(6):e03266-24. doi: 10.1128/spectrum.03266-24 (PMC12131755; doi:10.1128/spectrum.03266-24)
Supplement: Supplemental material — Legends. [file spectrum.03266-24-s0005.pdf]

Counting rare *Wolbachia* endosymbionts using digital droplet PCR

Alphaxand K. Njogu[1], Francesca Logozzo[1], William R. Conner[2], and J. Dylan Shropshire[1]

[1]Department of Biological Sciences, Lehigh University, Bethlehem, Pennsylvania, USA
[2]Division of Biological Sciences, University of Montana, Missoula, Montana, USA

Supplemental Legends

Table S1. *Wolbachia* genomes and accession numbers.

Table S2. *Drosophila* genomes and accession numbers.

Table S3. Statistical results.

Data S1. List of *ftsZ* sequences used to create ddPCR oligos in FASTA format.

Data S2. List of *mid1* sequences used to create ddPCR oligos in FASTA format.

Data S3. Data associated with figures.
